# Anti-herpetic Activity of *Macrocystis pyrifera* and *Durvillaea antarctica* Algae Extracts Against HSV-1 and HSV-2

**DOI:** 10.3389/fmicb.2020.02006

**Published:** 2020-09-11

**Authors:** Estefanía Castillo, Luisa F. Duarte, Nicolas Corrales, Diana M. Álvarez, Mónica A. Farías, Adolfo Henríquez, Patricio C. Smith, Cristian Agurto-Muñoz, Pablo A. González

**Affiliations:** ^1^ Millennium Institute on Immunology and Immunotherapy, Departamento de Genética Molecular y Microbiología, Facultad de Ciencias Biológicas, Pontificia Universidad Católica de Chile, Santiago, Chile; ^2^ GIBMAR, Grupo Interdisciplinario de Biotecnología Marina, Centro de Biotecnología, Universidad de Concepción, Concepción, Chile; ^3^ Escuela de Odontología, Facultad de Medicina, Pontificia Universidad Católica de Chile, Santiago, Chile; ^4^ Departamento de Ciencia y Tecnología de los Alimentos, Facultad de Farmacia, Universidad de Concepción, Concepción, Chile

**Keywords:** *Macrocystis pyrifera*, *Durvillaea antarctica*, HSV-1, HSV-2, antiviral activity, extract

## Abstract

Herpes simplex viruses (HSVs) type 1 (HSV-1) and type 2 (HSV-2) are highly prevalent in the human population, and the infections they produce are lifelong with frequent reactivations throughout life. Both viruses produce uncomfortable and sometimes painful lesions in the orofacial and genital areas, as well as herpetic gingivostomatitis, among other clinical manifestations. At present, the most common treatments against HSVs consist of nucleoside analogs that target the viral polymerases. However, such drugs are poorly effective for treating skin lesions, as they only reduce in 1–2 days the duration of the herpetic lesions. Additionally, viral isolates resistant to these drugs can emerge in immunosuppressed individuals, and second-line drugs for such variants are frequently accompanied by adverse effects requiring medical supervision. Thus, novel or improved therapeutic drugs for treating HSV lesions are needed. Here, we assessed the potential antiviral activity of aqueous extracts obtained from two brown macroalgae, namely *Macrocystis pyrifera* and *Durvillaea antarctica* against HSVs. Both extracts showed antiviral activity against acyclovir-sensitive and acyclovir-resistant HSV-1 and HSV-2. Our analyses show that there is a significant antiviral activity associated with proteins in the extract, although other compounds also seem to contribute to inhibiting the replication cycle of these viruses. Evaluation of the algae extracts as topical formulations in an animal model of HSV-1 skin infection significantly reduced the severity of the disease more than acyclovir, as well as the duration of the herpetic lesions, when compared to mock-treated animals, with the *D. antarctica* extract performing best. Taken together, these findings suggest that these algae extracts may be potential phytotherapeutics against HSVs and may be useful for the treatment and reduction of common herpetic manifestations in humans.

## Introduction

Herpes simplex viruses (HSVs) are highly prevalent in the human population, with herpes simplex virus type 1 (HSV-1) and herpes simplex virus type 2 (HSV-2) infecting approximately 70% (Looker et al., 2015) and 10% (Looker et al., 2008) of the world population, respectively. Importantly, infection with HSVs may elicit severe illnesses, such as ocular keratitis (corneal and stromal) potentially leading to permanent blindness, as well as life-threatening encephalitis in neonates and adults (Suazo et al., 2014; Duarte et al., 2019). However, the most common clinical manifestations related to HSV infections are ulcerous lesions in the skin, the orofacial area (*herpes labialis*), and genitalia (*herpes genitalis*), as well as inflammation of the gums (herpetic gingivostomatitis; Rechenchoski et al., 2017).

Acyclovir, valacyclovir, famciclovir, and penciclovir are nucleoside analogs commonly used for treating HSV infections (Kukhanova et al., 2014). These drugs target viral replication and differ from each other mainly in how they are processed into active nucleotide analogs, as well as their bioavailability in the body (Hasler-Nguyen et al., 2009; De Clercq, 2013; Sauerbrei, 2016). Although daily preventive oral intake of acyclovir reduces the shedding of HSV from both symptomatic and asymptomatic individuals, as well as the frequency of recurrent herpetic lesions in symptomatic persons, typical herpetic lesions still occur sporadically in these persons (Opstelten et al., 2008). On the other hand, therapeutic oral intake of acyclovir only reduces the time to loss of the scab in approximately 2 days (from 7.9 days in the placebo group to 5.8 days in the treated group), if taken early after the onset of the prodrome or erythema symptoms (Spruance and Freeman, 1990). Importantly, if acyclovir is taken during the papule stage, no significant therapeutic effects are observed (Spruance, 1993). Alternatively, when applied topically as a cream at a concentration of 5% (50 mg/ml), acyclovir only reduces the healing process in 1–2 days for *herpes labialis* (Fiddian and Ivanyl, 1983; Evans et al., 2002) and 3 days for *herpes genitalis* (Mertz et al., 1984; Leflore et al., 2000) of a total of 10 days in the placebo group (Moomaw et al., 2003; Arduino and Porter, 2006, 2008; Opstelten et al., 2008; Chen et al., 2016). Due to the modest effects elicited by acyclovir and other nucleoside analogs in treating herpetic skin lesions, the benefit provided by these antiviral drugs in reducing herpetic lesions has been questioned (Kroon et al., 1990; Mindel, 1991).

Importantly, acyclovir-resistant HSV isolates occur in 3.5–10% of immunosuppressed individuals, which obliges the use of second-line drugs that produce numerous adverse reactions and may require medical supervision (Ziyaeyan et al., 2007; Lolis et al., 2008; Suazo et al., 2014). In immunocompetent individuals, such drug-resistant variants occur at a lower frequency, yet approximately 1% of the cases (Bacon et al., 2002; Ziyaeyan et al., 2007). Given this scenario, new and more effective antivirals against HSVs are needed.

In the last decade, numerous alternatives for first‐ and second-line anti-HSV drugs have been investigated, and new drugs, such as viral helicase inhibitors are currently being tested in clinical trials (Chono et al., 2010; Wald et al., 2014). Numerous algae-derived compounds and botanicals with antiviral activity against HSV-1 have also been reported and tested in different studies. Some test the antiviral activity in experimental animal models (for a detailed review of botanical compounds with anti-HSV activity, please refer to Álvarez et al., 2020).

Thus, we sought to evaluate the potential antiviral properties of aqueous extracts obtained from two brown macroalgae, namely *Macrocystis pyrifera* and *Durvillaea antarctica*, against both HSV-1 and HSV-2 in a human cervix epithelial cell line (HeLa cells) and primary human gingival fibroblasts obtained from the oral cavity of healthy donors, as well as in a mouse model of HSV-1 skin infection. We found that both extracts display strong antiviral activity, some of which was significantly present in the protein fraction of the extracts suggesting that these algae extracts may have potential phytotherapeutic clinical applications for treating acyclovir-sensitive and acyclovir-resistant herpetic skin lesions.

## Materials and Methods

### Cell Cultures

Monolayers of HeLa cells (ATCC # CCL-2) were grown in culture dishes in DMEM growth media (Thermo Fisher Scientific), 5% FBS (Gibco, Thermo Fisher Scientific) supplemented with 1 mM pyruvate (Thermo Fisher Scientific), 2 mM glutamine (Thermo Fisher Scientific), and 100 IU/ml penicillin/streptomycin (Thermo Fisher Scientific) at 37°C and 5% CO_2_. Vero cells (ATCC # CCL-81) were grown in similarly with DMEM as described above. Primary cultures of human gingival fibroblasts derived from the tissue of the masticatory mucosa in the retromolar area of healthy adults (Institutional Ethical Committee approval #60823025) were generated using the explant method and grown in RPMI growth media (Thermo Fisher Scientific; Häkkinen and Larjava, 1992; Arancibia et al., 2009). Cell viability was determined for HeLa cells and gingival fibroblasts in black wall 96-well clear-bottom plates treated with algae extracts at increasing concentrations for 24 h and then incubated with alamarBlue® (resazurin, Thermo Fisher Scientific) at a 1:10 v/v ratio for 1 h at 37°C and measured in a Synergy Neo HTS Multi-Mode Reader (Biotek).

### Virus Preparation

Vero cells were used to propagate HSV-2 (333) ZAG, a recombinant virus that encodes green fluorescent protein (GFP) gene in its genome under the control of a CMV promoter (Wang et al., 2012), HSV-1 KOS (Macdonald et al., 2012), and HSV-1 K26-GFP, a recombinant virus that encodes GFP fused to the capsid protein VP26 (Desai and Person, 1998). To determine the titer of these viruses, supernatants and pellets of infected cells were collected and serially diluted over Vero cells in flat-bottom 96-well plates with Opti-MEM (Gibco, Thermo Fisher Scientific). Viral plaques (plaque-forming units, PFU) were counted 24 h post-infection (hpi). Supernatants and pellets obtained from uninfected Vero cells or gingival fibroblast cells were used as controls (mock infections). Acyclovir-resistant HSVs (ACV^R^-HSV-1 and ACV^R^-HSV-2) were obtained by propagating wild-type HSV viruses with increasing amounts of acyclovir (up to 200 μg/ml) until acyclovir-resistant viruses were obtained, as previously described (Sarisky et al., 2002; Ireland et al., 2016).

### Macroalgae Extract Preparations

*D. antarctica* and *M. pyrifera* macroalgae were collected at *Caleta Chome* in Chile (Latitude −36.772727°; Longitude −73.211766°) and thoroughly washed with water to eliminate salts and epiphyte organisms. The macroalgae were lyophilized for 5 days at −70°C using an Operon lyophilizer (FDU 7006, Operon), and then ground with a cast iron corn grinder and passed through a 0.5 mm mesh (U.S. Standard Sieve Series, Dual Manufacturing). Three grams of lyophilized alga powder was resuspended in 120 ml of deionized water and agitated for 4 h at 200 RPM and 30°C. The preparation was then centrifuged at 4,000 RPM for 10 min, and the supernatant recovered and incubated overnight with 96% ethanol at 4°C (Droguería Diprolab) at a 1:1 proportion to precipitate alginates in the extract. The preparation was then centrifuged at 4,000 RPM for 10 min (Rotofix 32 A, Hettich) to separate the precipitated alginates from the soluble fraction, which was then filtered through a 0.45 μm filter. The ethanol was removed from the supernatant by evaporation (Multivapor™ P-6/P-12, Büchi), and the remaining solution lyophilized at −70°C until freeze-dried. A stock solution of the algae extracts was prepared at 125 mg/ml after lyophilization with deionized water and kept at −80°C. Proteins in the algae extracts were precipitated with 100% w/v ammonium sulfate (Emsure®, MercK KGaA) overnight at 4°C, and then, the suspensions were centrifuged at 20,000 *g* at 4°C. The supernatants were dialyzed using a 3.5 kDa MWCO dialysis membrane (Spectra/Por® 3, Spectrum Laboratories) and 2 L of deionized water, which was replaced five times in 10 h. The protein pellets were washed with deionized water and then dialyzed as mentioned for the supernatants. The remaining solution was lyophilized at −70°C until freeze-dried, and a stock solution was prepared at 125 mg/ml using PBS 5X. To generate the size-fractionated algae extracts, Amicon® Ultra (Merck Millipore) centrifugal filtering devices with a 10 kDa cut-off pore were used. Ten milliliters (ml) of each alga extract was added to the filtering device and centrifuged at 4,000 × *g* for 30 min at 4°C. The filtered fraction, enriched in compounds smaller than 10 kDa, was labeled “<10 kDa,” while the fraction remaining in the filtering device enriched in compounds over 10 kDa in size was labeled “>10 kDa.”

### Cytotoxicity Assay

For the cytotoxicity assay, HeLa cells and human gingival fibroblasts were seeded on to 96-well plates, incubated with decreasing concentrations of the algae extracts for 24 h starting at 125 mg/ml and then treated to assess the cell viability using a resazurin-based assay (alamarBlue®, Thermo) according to manufacturer’s instructions. The 50% cytotoxic concentration (CC_50_), corresponding to the concentration of alga extract that reduces 50% of the viability of the cells, was calculated by performing a nonlinear regression analysis to generate a four-parameter sigmoid dose-response curve using Prism software (GraphPad, La Jolla California USA).

### Macroalgae Extract Treatments and Infections

About, 1.5 × 10^5^ cells/well in 24-well plates were infected with the amounts of viruses indicated in the figure legends for 1 h in Opti-MEM. Past this time, supernatants were replaced with fresh Opti-MEM and complemented with varying concentrations of the algae extracts, as indicated in each figure. For the plaque reduction assays, cells were infected with a fixed amount of PFUs of GFP-encoding HSV-1 (K26-GFP) or HSV-2 (333) ZAG and treated with decreasing non-toxic concentrations of the algae extracts or acyclovir at 50 μg/ml. Fluorescent-focus units (FFUs), which are virus-infected fluorescent cells that will become viral plaques after some time, were quantified using a fluorescence microscope 24 hpi (AxioVert.A1, Zeiss). The percentage of inhibition of the algae extracts was calculated based on the number of FFUs counted in the presence and absence of the extracts or acyclovir as follows: [1 − (FFUs in the presence of the algae extracts or acyclovir/FFUs in the mock)] × 100. The 50% effective concentration (EC_50_) of each extract, defined as the concentration of extract required to reduce the yield of FFUs by 50%, was determined using a nonlinear regression analysis using Prism software (GraphPad, La Jolla California USA). As indicated in the text and figure legends, the cells were incubated with acyclovir starting 1 h after infection for a total time of 24 h or with the algae extracts either, for 1 h previous to infection and then removed from the culture (Pre), for 6 h after infection and then removed from the culture (Post), or 1 h previous to infection and then added again after infection for 6 h (Continuous treatment). For the viral titer assays carried out with the fractionated algae extracts, HeLa cells were incubated with a final concentration of 1 mg/ml of the fractionated algae extracts. In these experiments, a pre-treatment protocol (Pre) was used for the *M. pyrifera* fractioned extract. In contrast, a post-treatment protocol (Post) was used for the *D. antarctica* fractioned extract.

### HSV-Binding Assay in HeLa Cells

Cells were treated with vehicle (Opti-MEM alone), *M. pyrifera* or *D. antarctica* algae extracts (1 mg/ml) or acyclovir (50 μg/ml) in Opti-MEM for 1 h. After this time, HeLa cells were incubated at 4°C for 15 min and subsequently infected with HSV-2 (333) ZAG or HSV-1 K26-GFP viruses at different multiplicity of infections (MOIs; 1.0, 0.5, 0.1, and 0.05). Then, the cells were maintained at 4°C for 5 h. Past this time, HeLa cells were washed three times with PBS 1X, released from the plates with a cell scraper, and centrifuged at 3,200 RPM for 5 min. The cell pellets were stored at −80°C until protein extraction. The protein extracts were processed to detect gB and gD viral protein expression levels by western blot analyses.

### Western Blots

Protein samples were prepared using RIPA buffer and quantified using the Pierce BCA Protein Assay Kit (Thermo). Western blot membranes were blocked with 5% skim milk and incubated with either of the following antibodies: 1:50,000 anti-gD monoclonal antibody (Virusys, clone HA025), 1:1,000 anti-gB monoclonal antibody (Virusys, clone 10B7), or 1:1,500 anti-β-actin (Biolegend, clone 2F1-1). For the detection step, membranes were incubated with an anti-mouse-IgG HRP-conjugated polyclonal antibody (GenScript) and then incubated with a luminol:coumaric acid solution. Chemiluminescence was visualized using a ChemiDoc®MP Imaging System (Bio-Rad), and the intensity of the bands was determined using ImageJ software (NIH, USA).

### Quantitative PCR

Samples were processed at the indicated time-points post-infection and processed for DNA extraction using the phenol-chloroform method as previously described (Retamal-Díaz et al., 2017). Total DNA was used for quantitative PCR (qPCR) analysis using 200 ng of DNA per reaction with the following primers and probe for the *UL30* gene: Fwd-GGCCAGGCGCTTGTTGGTGTA, Rev-ATCACCGACCCGGAGAGGGA, and Probe CCGCCGAACTGAGCAGACACCCGC and an Applied Biosystems StepOnePlus thermocycler (Petro et al., 2015; Retamal-Díaz et al., 2017).

### *In vivo* Experiments

Mice were obtained from the Jackson Laboratories (Bar Harbor) and maintained at the animal facility at the Pontificia Universidad Católica de Chile. All procedures were approved by the Ethical Committee for Animal and Environmental Care (protocol #170416002). About 4–6 weeks-old C57BL/6 female mice were anesthetized with ketamine-xylazine. The right flank of each mouse was depilated and shaved using VEET®. The next day, the epidermal layer of skin was exposed by abrasion with a nail file and infected with 10^6^ PFU of HSV-1 (KOS) in 10 μl and allowed to dry. Mock-infected mice were used as controls. Treatments using algae extract preparations at 5 mg/ml were started 24 hpi and applied twice daily (8 AM and 6 PM) for 10 days. The algae extracts were formulated in 2% hydroxymethyl cellulose (Reccius). Topical 5% acyclovir (Mintlab) and vehicle treatments were used as controls. The epithelial disease was scored as follows: (1) erythema or primary lesion, (2) slight erythema/edema and distant zosteriform lesions, (3) ulceration and edema, increased epidermal spread, and (4) hind-limb paralysis. Skins and dorsal root ganglia were removed to determine viral titers over Vero cells and viral genome loads by qPCR at 5 d.p.i. Viral genome loads were determined as described above.

### Statistical Analysis

Statistical significance between experimental groups was assessed by one-way or two-way analysis of variance (ANOVA) with Dunnett’s multiple comparison test (*in vitro* experiments) or Bonferroni’s post-test (*in vivo* experiments) using GraphPad Prism (GraphPad Software, La Jolla California USA) as indicated in the figure legends.

## Results

### *Macrocystis pyrifera* and *Durvillaea antarctica* Aqueous Extracts Interfere With the Replication Cycle of HSV

**Figure 1:**
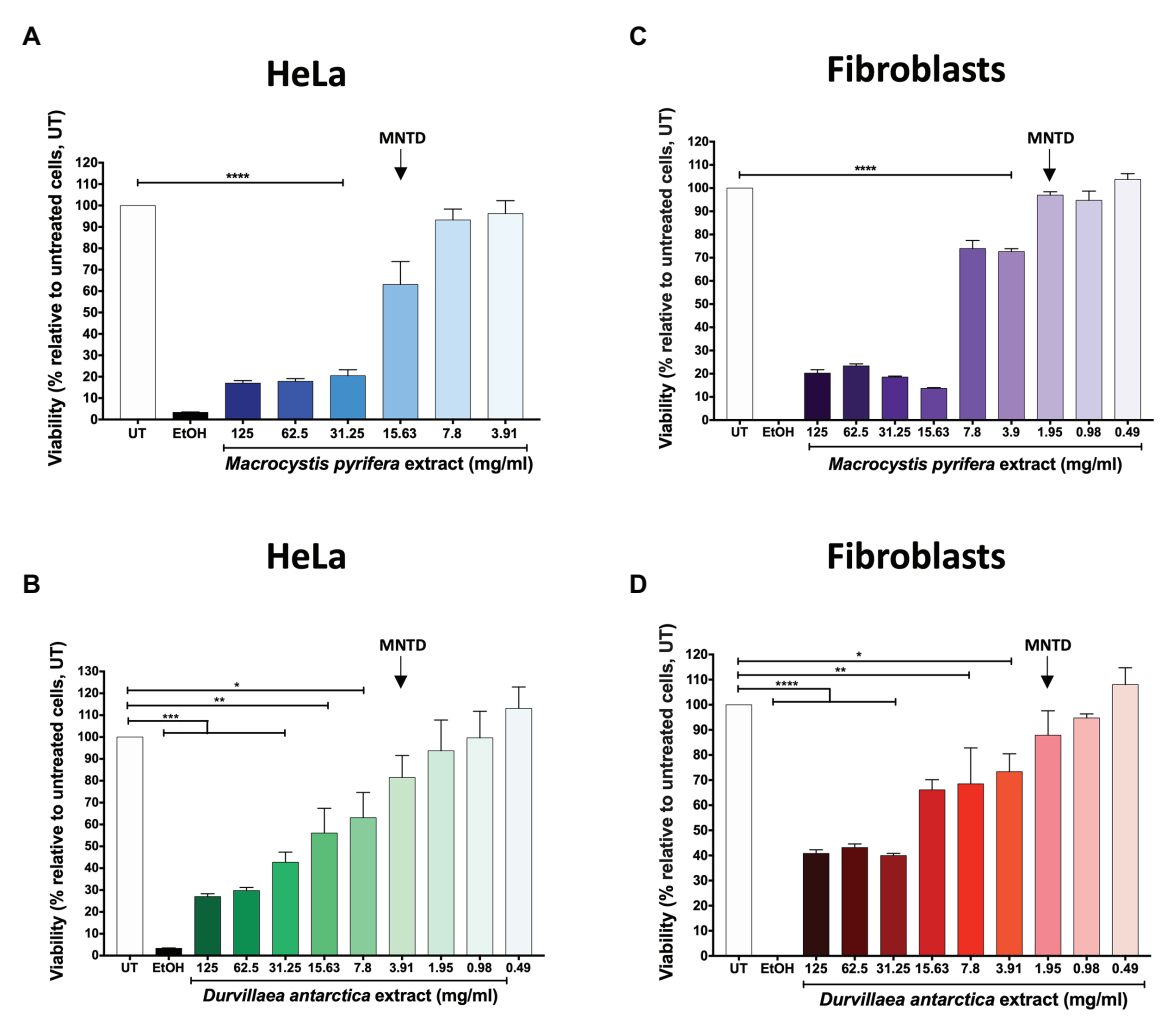
Maximum non-toxic dose (MNTD) of the *Macrocystis pyrifera* and *Durvillaea antarctica* aqueous extracts. HeLa cells and primary culture of gingival fibroblasts were incubated with serially-diluted aqueous extracts of *M. pyrifera* and *D. antarctica* for 24 h, starting with 125 mg/ml. Cell viability was evaluated at 24 h post-treatment using a resazurin-based assay (alamarBlue®). The MNTD is defined as the maximum concentration of extract that does not significantly alter cell viability *in vitro* when compared to untreated cells. **(A)** MNTD for HeLa cells treated with the *M. pyrifera* extract. **(B)** MNTD for HeLa cells treated with the *D. antarctica* extract. **(C)** MNTD for human gingival fibroblasts treated with the *M. pyrifera* extract. **(D)** MNTD for human gingival fibroblasts treated with the *D. antarctica* extract. UT is untreated cells, and EtOH represents cells treated with 70% ethanol. Data shown are means ± standard error deviation (SEM) of three independent experiments. The data were analyzed using one-way ANOVA and Dunnett’s multiple comparisons test. ^****^p < 0.0001; ^***^p < 0.001; ^**^p < 0.01; and ^*^p < 0.05.

In this study, we evaluated the potential antiviral activity of aqueous extracts obtained from two brown macroalgae, namely *M. pyrifera* and *D. antarctica*, against HSV-1 and HSV-2 both, *in vitro* and *in vivo*. Because aqueous extracts of *M. pyrifera* and *D. antarctica* have gel-like consistencies that make their manipulation challenging and the preparation of precise dilutions difficult, we removed the carbohydrate polymers in the extracts by ethanol precipitation. Then, before assessing the potential antiviral effects of these extracts, we assessed if these preparations were toxic or not for the cell cultures and determined the maximum non-toxic dose (MNTD), as well as the 50% cytotoxic concentration (CC_50_) starting with a concentration of 125 mg/ml and 2-fold serial dilutions. The CC_50_ is defined as the concentration of alga extract that causes a reduction in 50% of the cell viability. As shown in Figures 1A–D, the MNTD for the *M. pyrifera* and *D. antarctica* extracts was 15.6 and 3.9 mg/ml in HeLa cells and 1.95 mg/ml in primary cultures of fibroblasts for both extracts. The CC_50_ values for the *M. pyrifera* and *D. antarctica* extracts were 18.2 and 17.2 mg/ml in HeLa cells and 9.2 and 24.9 mg/ml in primary cultures of fibroblasts (Supplementary Figures 1A–D).

**Figure 2:**
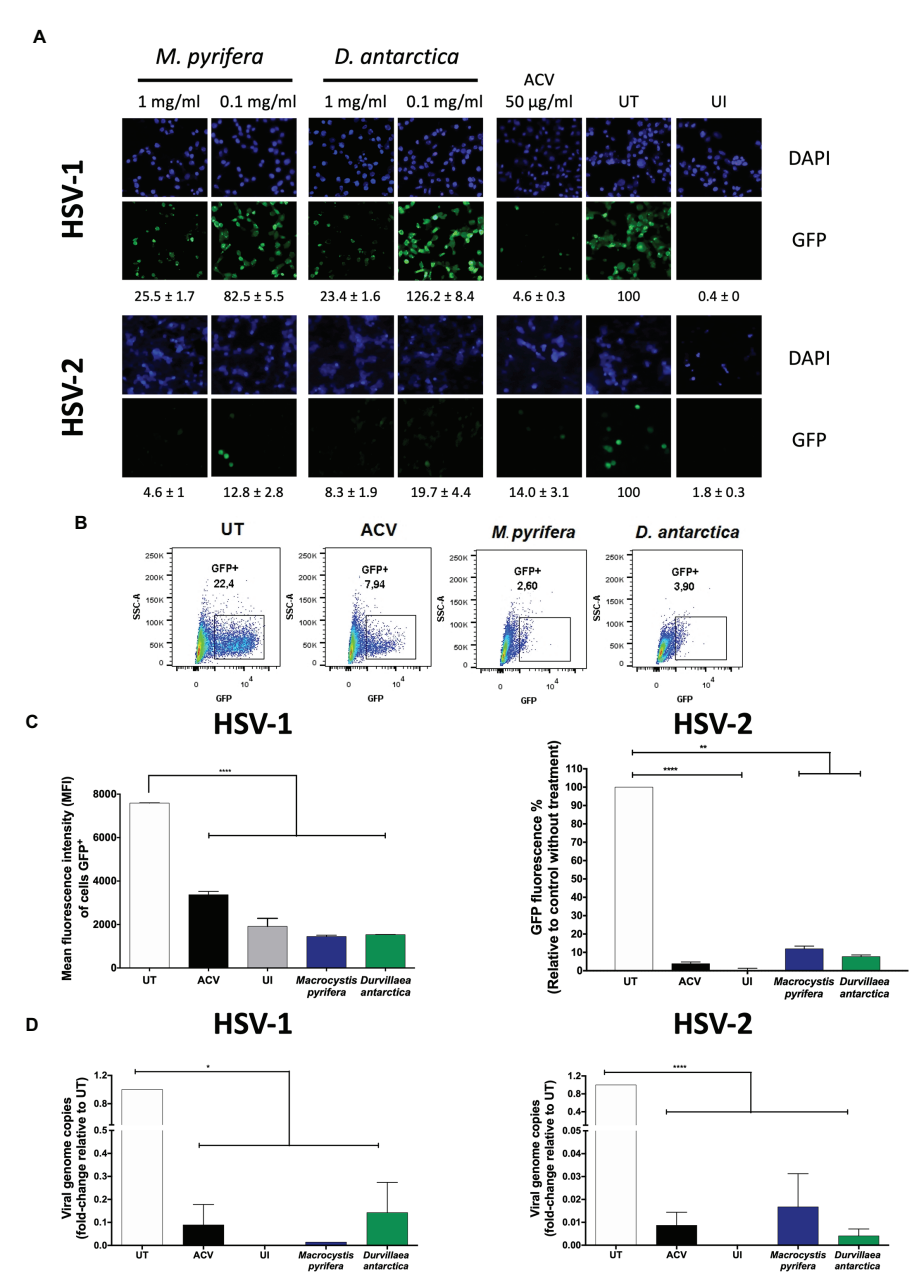
*In vitro* herpes simplex virus (HSV) antiviral activity of the *M. pyrifera* and *D. antarctica* algae extracts. HeLa cells were pre-treated 1 h with the aqueous extracts, then infected with HSV at an multiplicity of infection (MOI) 1, and 1 h post-infection (hpi) treated again with the algae extracts either, for 24 h [virus-derived green fluorescent protein (GFP) fluorescence] or 12 h (virus replication). As a control, acyclovir (ACV, 50 μg/ml) was applied immediately after infection and left for the complete duration of the assay. **(A)** Representative fluorescence microscopy images of virus-derived GFP fluorescence (green) in cells treated with the *M. pyrifera* or *D. antarctica* algae extracts at 1 or 0.1 mg/ml and infected with HSV-1 (upper set of panels) or HSV-2 (lower set of panels). The extracts were present during the complete duration of the assay after infection. Cell nuclei were counterstained with DAPI (blue). GFP-derived fluorescence was quantified using ImageJ software. Data are means ± SEM of three different image fields. Representative images are shown. **(B)** Representative FACS plots showing the frequency of GFP-positive cells infected with HSV-1 and treated with the algae extracts. ACV, acyclovir; UT, untreated. **(C)** Left: mean fluorescence intensity of GFP-positive cells infected with HSV-1 (determined by flow cytometry 24 hpi) treated with *M. pyrifera* (blue bars) or *D. antarctica* (green bars) at 1 mg/ml for the complete duration of the assay after infection. Right: GFP fluorescence of HSV-2-infected cells (determined with a multi-mode reader cytometry 24 hpi) treated with the *M. pyrifera* (blue bars) or *D. antarctica* (green bars) extracts at 1 mg/ml for the complete duration of the assay after infection. **(D)** Viral genome copies (*UL30* gene) were determined by quantitative PCR (qPCR) 12 hpi in cells infected with HSV-1 (left graph) or HSV-2 (right graph) at an MOI 1 and treated with the *M. pyrifera* (blue bars) or *D. antarctica* (green bars) extracts at 1 mg/ml for the complete duration of the assay after infection. UT, untreated cells; UI, uninfected cells. Data shown correspond to means ± SEM of three independent experiments for **(A–C)** and two experiments for **(D)**. The data were analyzed using one-way ANOVA and Dunnett’s multiple comparisons test. ^****^p < 0.0001; ^***^p < 0.001; ^**^p < 0.01; and ^*^p < 0.05.

As a preliminary approach, HeLa cells were treated for 1 h with the algae extracts at 0.1 or 1 mg/ml previous to infection, then inoculated for 1 h with GFP-encoding HSV viruses at an MOI 1, and incubated again with the algae extracts for the complete duration of the assay. GFP fluorescence and viral DNA analyses were performed 24 and 12 h after infection, respectively. As shown in Figure 2A, under a fluorescence microscope, cells treated with the algae extracts showed a pronounced reduction in HSV-derived GFP fluorescence. This effect was more pronounced with 1 mg/ml of the extract than 0.1 mg/ml (Figure 2A). Based on these results, we then quantified 24 h after infection the amount of GFP-derived fluorescence in HSV-infected cells treated with 1 mg/ml of each of the extracts either by flow cytometry for HSV-1 or with a multi-mode plate reader for HSV-2 (Figures 2B,C). Importantly, cells treated with the algae extracts displayed reduced GFP-derived fluorescence. Additionally, these treatments significantly reduced the amount of HSV-1 and HSV-2 viral genome copies in the infected cells at 12 hpi (Figure 2D).

**Figure 3:**
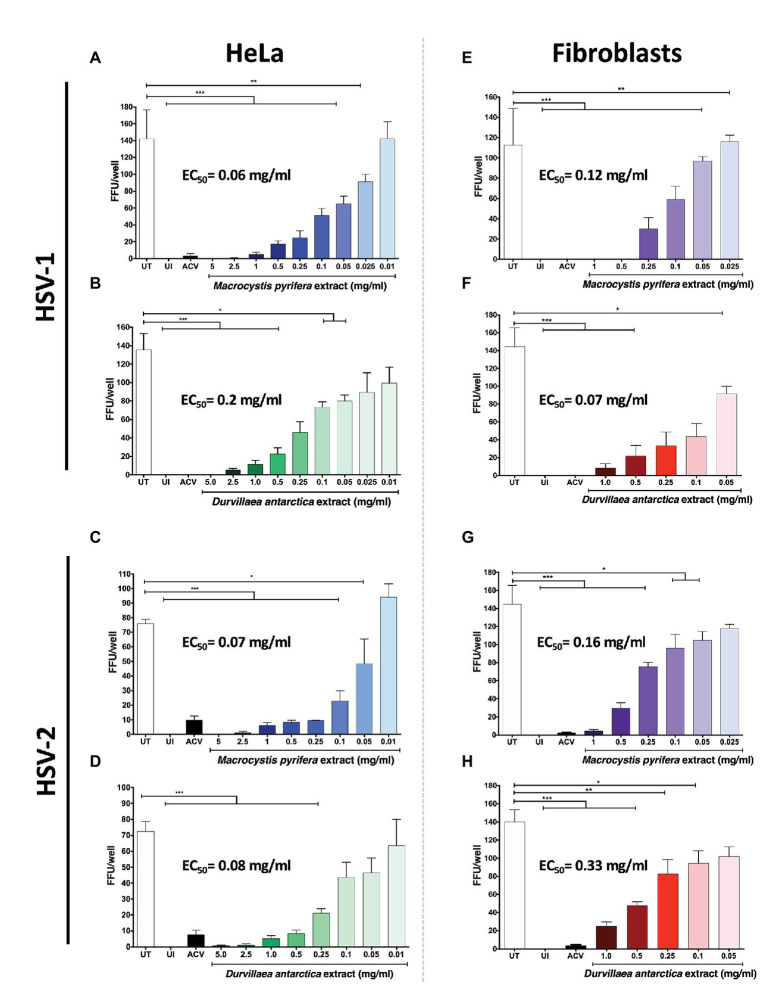
Effective concentration of the *M. pyrifera* and *D. antarctica* algae aqueous extracts against HSV. Cells were infected with a fixed amount of PFUs of GFP-encoding HSV-1 or HSV-2 obtained from serial dilutions of viral stocks. Then, cells were treated with decreasing non-toxic concentrations of the algae extracts for the remaining time of the assay, or acyclovir (ACV) at 50 μg/ml. The 50% effective concentration (EC_50_) was determined by quantifying fluorescent-focus units (FFUs) using a fluorescence microscope 24 hpi and is defined as the concentration of algae extract that reduces in 50% the number of FFUs compared to untreated (UT) cells. UI: uninfected cells. **(A,B)** EC_50_ of *M. pyrifera* and *D. antarctica* extracts against HSV-1 infection in HeLa cells. **(C,D)** EC_50_ of *M. pyrifera* and *D. antarctica* extracts against HSV-2 infection in HeLa cells. **(E,F)** EC_50_ of *M. pyrifera* and *D. antarctica* against HSV-1 infection in human gingival fibroblasts. **(G,H)** EC_50_ of *M. pyrifera* and *D. antarctica* against HSV-2 infection in human gingival fibroblasts. Data shown are means ± SEM of three independent experiments. The data were analyzed using one-way ANOVA and Dunnett’s multiple comparisons tests. ^***^p < 0.001; ^**^p < 0.01; and ^*^p < 0.05.

Next, we sought to determine the 50% effective concentrations (EC_50_) of the algae extracts by using an assay that counts the amount of FFUs generated in HeLa cells and gingival fibroblasts. The EC_50_ is the concentration of alga extract that causes a reduction in 50% of FFUs. Cells infected with a fixed amount of PFUs of the GFP-encoding HSVs and treated with the algae extracts displayed a reduction of HSV-1 (Figures 3A,B) and HSV-2 (Figures 3C,D) FFUs in HeLa cells with EC_50_ values of 0.06 and 0.07 mg/ml for *M. pyrifera* and 0.2 and 0.08 mg/ml for *D. antarctica*, respectively. A similar effect was observed with the gingival fibroblasts infected with HSV-1 and HSV-2, with EC_50_ values of 0.12 and 0.16 mg/ml with the *M. pyrifera* extract (Figures 3E,G), and 0.07 and 0.33 mg/ml for the *D. antarctica* extract (Figures 3F,H), suggesting potential applications for these extracts in treating skin and oral herpetic clinical manifestations.

**Figure 4:**
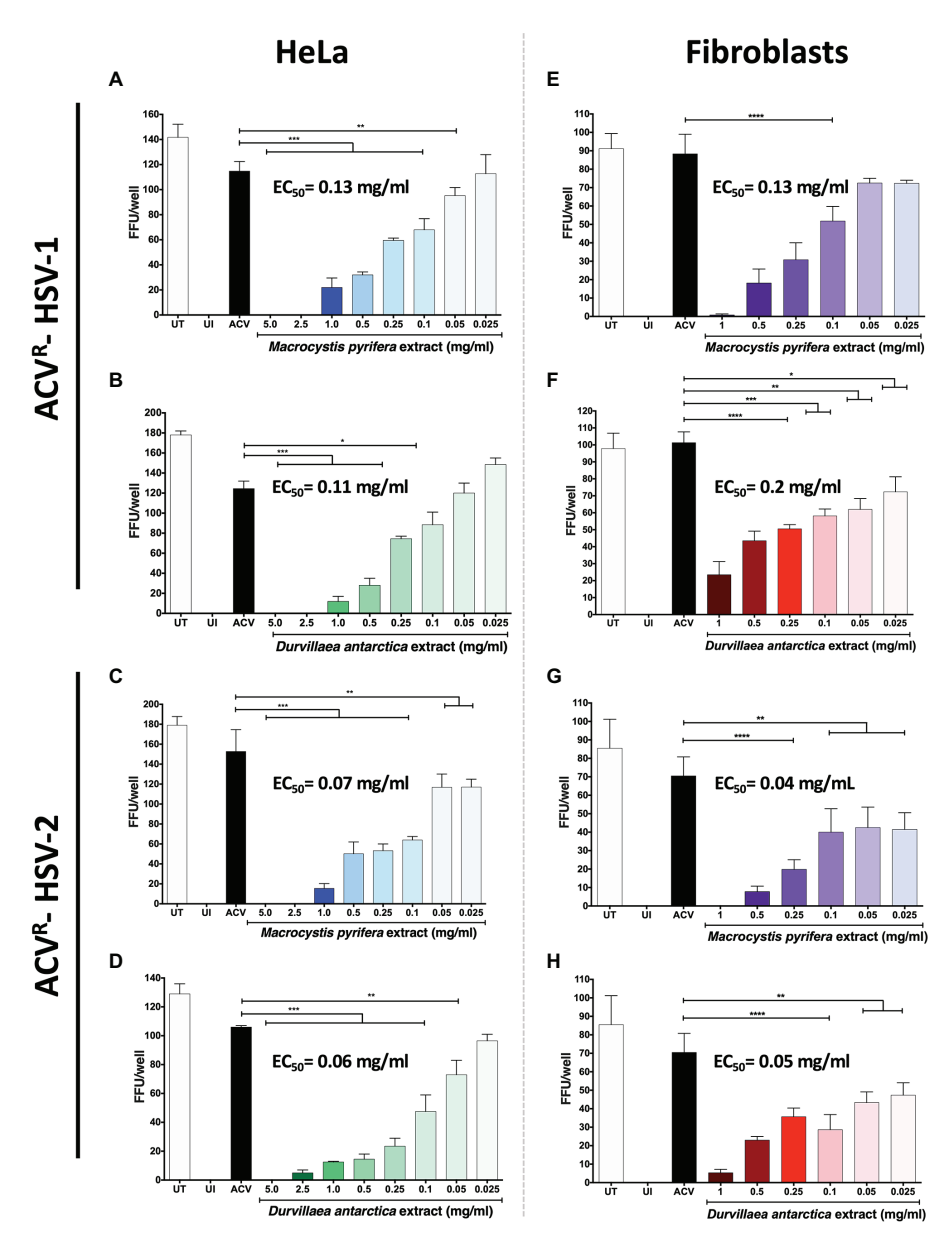
Antiviral activity of the *M. pyrifera* and *D. antarctica* algae aqueous extracts against acyclovir-resistant (ACV^R^) HSV. Cells were infected with a fixed amount of PFUs of acyclovir-resistant HSV-1 or HSV-2 and treated with decreasing non-toxic concentrations of the algae extracts or acyclovir at 50 μg/ml for the complete duration of the assay after infection. The 50% effective concentration (EC_50_) was determined by quantifying fluorescent-focus units (FFUs) using a fluorescence microscope 24 hpi. **(A,B)** FFUs in HeLa cells infected with ACV^R^-HSV-1 and treated with the *M. pyrifera* or *D. antarctica* extracts. **(C,D)** FFU in HeLa cells infected with ACV^R^-HSV-2 and treated with the *M. pyrifera* or *D. antarctica* extracts. **(E,F)** FFUs in human fibroblasts infected with ACV^R^-HSV-1 and treated with the *M. pyrifera* or *D. antarctica* extracts. **(G,H)** FFUs in human fibroblasts infected with ACV^R^-HSV-2 and treated with the *M. pyrifera* or *D. antarctica* extracts. Data shown are means ± SEM of three independent experiments. The data were analyzed using one-way ANOVA and Dunnett’s multiple comparisons tests. ^****^p < 0.0001; ^***^p < 0.001; ^**^p < 0.01; and ^*^p < 0.05.

To assess whether the antiviral effects observed above also applied to acyclovir-resistant HSVs, we tested the capacity of these extracts to interfere with the propagation of acyclovir-resistant HSV-1 and HSV-2 (ACV^R^-HSV). As shown in Figure 4, both extracts displayed antiviral activity against ACV^R^-HSV-1 (Figures 4A,B) and ACV^R^-HSV-2 (Figures 4C,D), in HeLa and fibroblast cells (Figures 4E–H), similar to the antiviral activities observed against acyclovir-sensitive HSVs. These results overall indicate that the antiviral mechanism of action of these extracts differs from that of acyclovir and, thus could eventually be used to treat infections by such acyclovir-resistant variants.

### The Antiviral Activity of the Algae Extracts Is Effective When Applied Previously and After Infection

**Figure 5:**
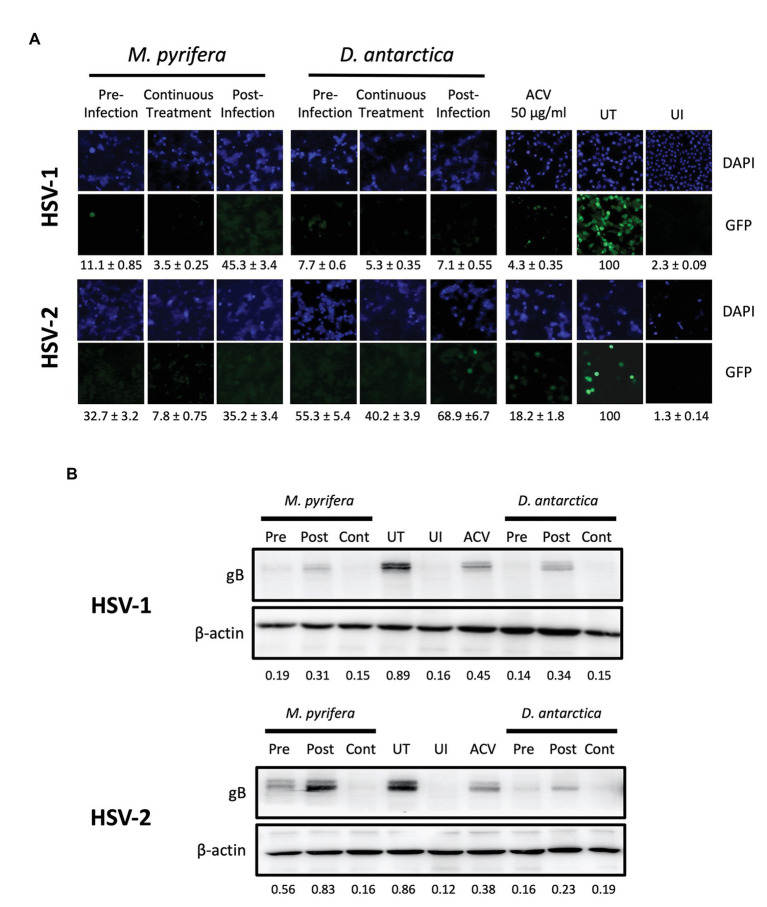
Antiviral activity of the *M. pyrifera* and *D. antarctica* algae aqueous extracts added at different time-points relative to HSV infection to HeLa cells. The algae extracts were added at 1 mg/ml to HeLa cells at different time-points relative to infection. The treatments with the algae extracts were added either, 1 h previous to infection and then removed after infection (Pre-infection), added 1 h after infection and left for a total of 6 h (Post-infection), or added in a continuous manner, that is, 1 h before infection and then after infection for 6 h (continuous treatment). Cells were infected with HSV at an MOI 1. **(A)** Representative images of GFP-derived fluorescence (green) in cells treated either, with the *M. pyrifera* or *D. antarctica* extracts and infected with a GFP-encoding HSV-1 (upper panels) or GFP-encoding HSV-2 (lower panels). Cell nuclei were counterstained with DAPI (blue). GFP-derived fluorescence was quantified using ImageJ software. **(B)** Western blot analyses for the viral protein gB and host protein beta-actin (loading control) 24 hpi with HSV-1 (upper panels) or HSV-2 (lower panels). Representative images are shown for the western blots. The quantifications correspond to the means of three independent experiments. The GFP fluorescence and the intensity of the bands in the western blots were quantified using ImageJ software. UI, uninfected; UT, untreated; ACV, acyclovir.

To better understand how the algae extracts interfere with the replication cycles of HSV-1 and HSV-2, we added the extracts at different time-points relative to the infection of the cell cultures and then assessed the virus-derived GFP fluorescence and viral protein expression levels in the cells. To evaluate this, the algae extracts were either added to HeLa cultures 1 h previous to infection and then removed immediately before infection (Pre), applied 1 h after infection and left in the culture for a total of 6 h (Post) in which case the supernatants were replaced after this time with fresh Opti-MEM or added 1 h before infection and then again right after infection for 6 h (Continuous treatment). In this latter case, the supernatants were also replaced after 6 h with fresh Opti-MEM. As a control, acyclovir (ACV) was added to the cells 1 hpi for 24 h at a final concentration of 50 μg/ml. As shown in Figure 5A, fluorescence microscopy analyses showed that the treatments with both extracts, added either before infection, after infection or in a continuous manner, were effective in reducing virus-derived GFP expression for both HSV-1 and HSV-2. However, the post-infection treatment was seemingly slightly less effective in some cases compared to the other treatments, as some viral protein expression was observed with this treatment by western blot in HeLa cells infected with HSV-1 or HSV-2 (Figure 5B). Nevertheless, a significant and evident antiviral effect was observed under this condition, which was evidenced in the fluorescence microscopy assay.

**Figure 6:**
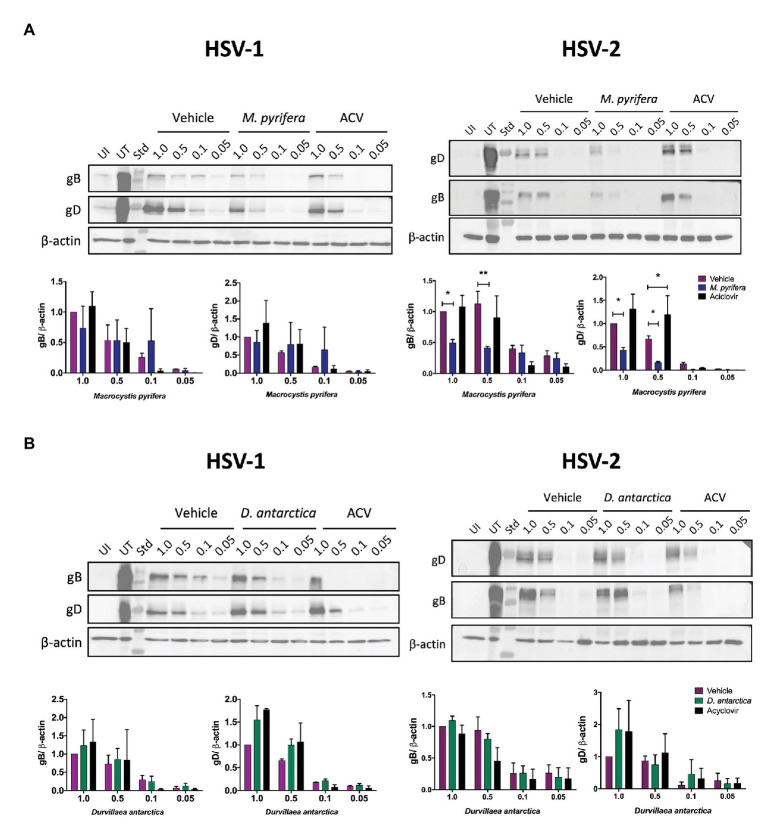
HSV binding to the surface of HeLa cells in the presence of the algae extracts. HeLa cells were treated with the *M. pyrifera* or *D. antarctica* extracts for 1 h. Then, the cells were incubated at 4°C for 15 min and later infected with different MOIs (1, 0.5, 0.1, or 0.05) of HSV-1 (left panels) or HSV-2 (right panels) for 5 h at 4°C, washed with saline buffer and immediately processed for protein extraction. **(A)** Western blot analyses of the viral proteins gB and gD in HeLa cells treated with the *M. pyrifera* extract or **(B)** the *D. antarctica* extract. Representative images are shown for the western blots. The quantifications were normalized relative to the vehicle and virus at an MOI 1 and corresponded to means ± SEM of two independent experiments for HSV-1 and three independent experiments for HSV-2. The intensity of the bands were quantified using ImageJ software The data were analyzed using two-way ANOVA and Dunnett’s multiple comparisons tests. ^**^p < 0.01; ^*^p < 0.05. UI, uninfected; UT, untreated; ACV, acyclovir; Std, standard.

To assess the possibility that the algae extracts block the ability of the viruses to bind to the cell surface, we evaluated the amount of HSV particles bound to the surface of HeLa cells inoculated with the viruses at 4°C, by determining the amount of glycoprotein B and glycoprotein D present in the cells after an incubation period of 5 h at this temperature (Ibáñez et al., 2018). As shown in Figure 6A, the treatment with the *M. pyrifera* extract decreased the amount of HSV-2 bound to the cell surface. For the treatments with the *D. antarctica* extract, no significant variations were observed regarding the binding of HSVs to the cell surface (Figure 6B). Overall, these results indicate that, in most cases, the extracts are acting at one or more steps that are downstream of the virus binding to the cell surface, except for the *M. pyrifera* extract and HSV-2.

### Significant Antiviral Activity Is Concentrated in the Protein Fraction of Algae Extracts

**Figure 7:**
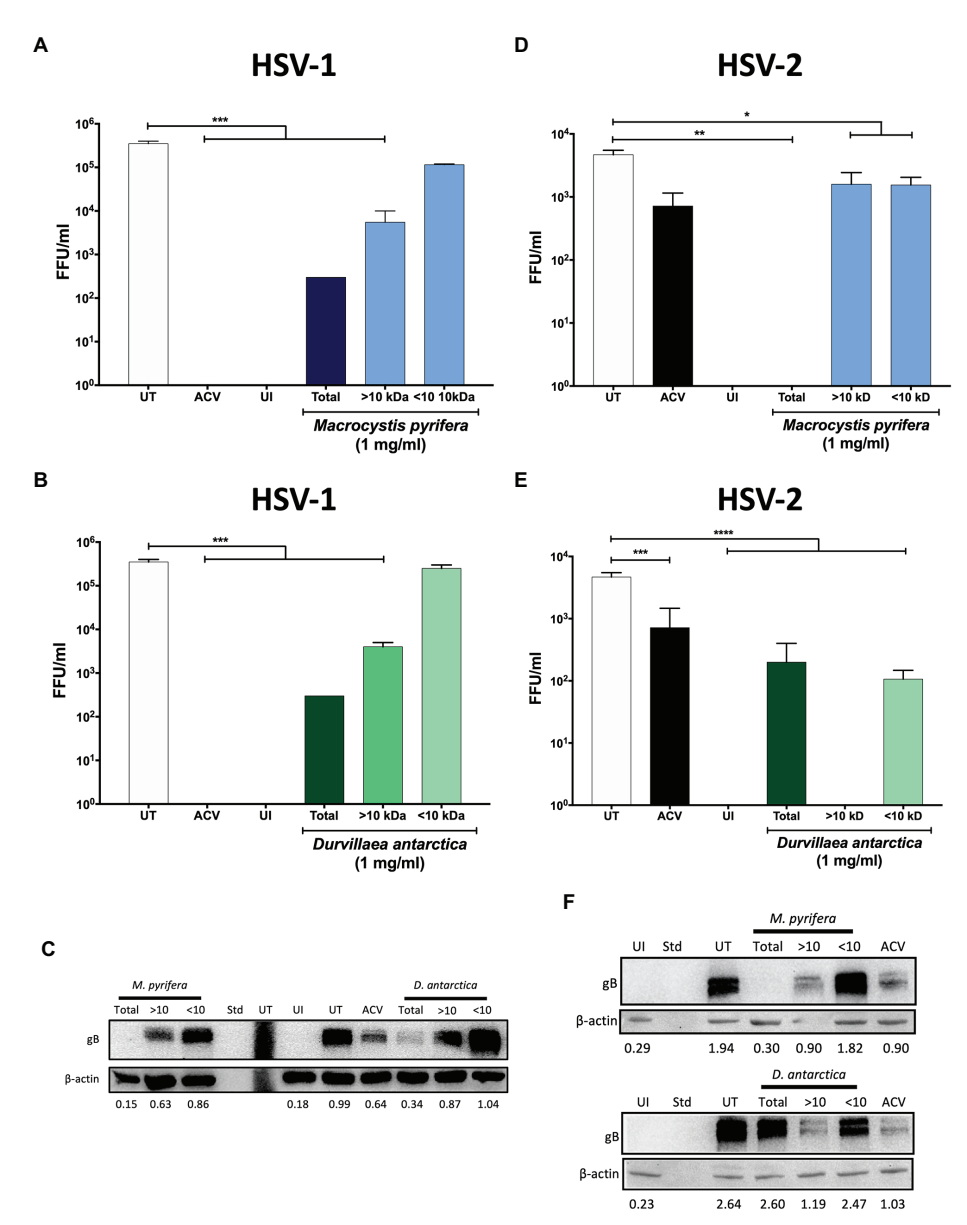
Antiviral activity in *M. pyrifera* and *D. antarctica* algae extracts fractionated by their size. The algae extracts were processed to separate >10 kDa or <10 kDa compounds. HeLa cells were infected with GFP-encoding HSV-1 or HSV-2 at an MOI 1 and then treated with 1 mg/ml of the >10 kDa algae fraction, <10 kDa algae fraction, or total extract. **(A)** Viral titers obtained from the supernatants of HSV-1-infected cells treated with the *M. pyrifera* extract for 24 h. **(B)** Viral titers obtained from the supernatants of HSV-1-infected cells treated with the *D. antarctica* extract 24 h. **(C)** Western blot analyses of the viral protein gB and the host protein beta-actin (loading control) 24 hpi with HSV-1 in cells treated with either antarctica for 24 h. **(D)** Viral titers obtained from the supernatants of HSV-2-infected cells treated with the *M. pyrifera* extract for 24 h. **(E)** Viral titers obtained from the supernatants of HSV-2-infected cells treated with the *D. antartica* extract for 24 h. **(F)** Western blot analyses of the viral protein gB and the host protein beta-actin (loading control) 24 hpi with HSV-2 in cells treated with either extract for 24 h. Representative images are shown for the western blots. Data shown are means ± SEM of three independent experiments. The data were analyzed using one-way ANOVA and Dunnett’s multiple comparisons tests. ^****^p < 0.0001; ^***^p < 0.001; ^**^p < 0.01; and ^*^p < 0.05. UI, uninfected cells; UT, untreated cells; ACV, acyclovir-treated cells; Std, standard.

**Figure 8:**
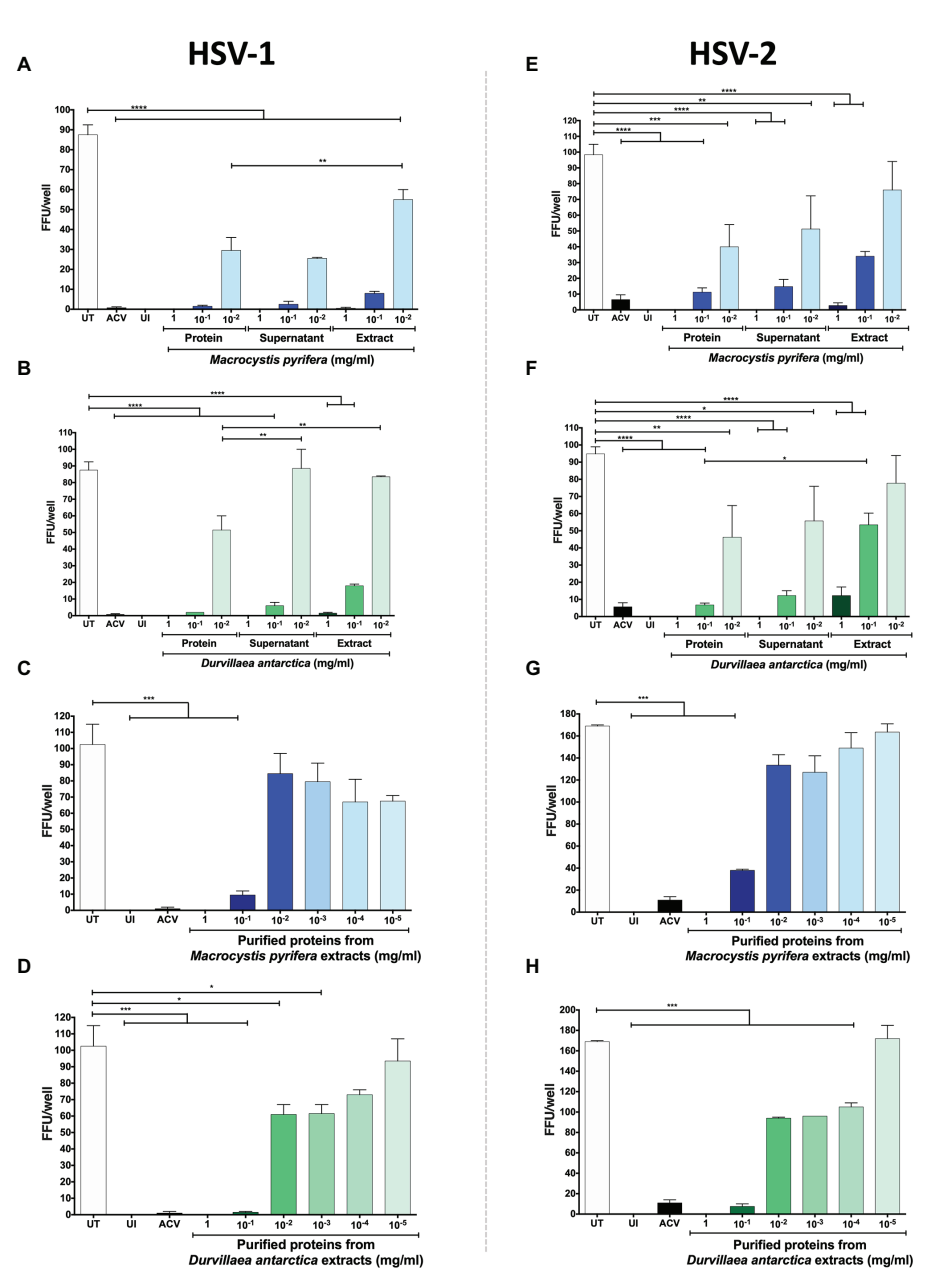
HSV antiviral activity of the protein fraction of the *M. pyrifera* and *D. antarctica* algae extracts. The algae aqueous extracts were processed to separate the proteins in the algae extracts. HeLa cells were infected a fixed amount of PFUs of GFP-encoding HSV-1 or HSV-2 at an MOI 1 and treated with serial dilutions of either of the algae protein fractions, supernatants or total extracts for 24 h. FFUs in all panels were determined at 24 hpi. **(A,B)** HSV-1-infected cells treated with serial dilutions of the precipitated proteins of the *M. pyrifera* or *D. antarctica* extracts, supernatants of the precipitated proteins or the total extract. **(C,D)** HSV-1-infected cells treated with serial dilutions of the precipitated proteins obtained from the *M. pyrifera* or *D. antarctica* extracts. **(E,F)** HSV-2-infected cells treated with serial dilutions of the precipitated protein of the *M. pyrifera* or *D. antarctica* extracts, supernatants of the precipitated proteins, or the total extract. **(G,H)** HSV-2-infected cells treated with serial dilutions of the precipitated proteins obtained from the *M. pyrifera* or *D. antarctica* extracts. Data shown are means ± SEM of three independent experiments. The data were analyzed using one-way ANOVA and Dunnett’s multiple comparisons tests. ^****^p < 0.0001; ^***^p < 0.001; ^**^p < 0.01; and ^*^p < 0.05.

To narrow down the properties of the antiviral compounds present in the algae preparations, we fractioned the extracts based on their size using centrifugal filtering devices with a 10 kDa cut-off pore. As shown in Figures 7A,B, the antiviral activity of the extracts was generally in the >10 kDa fractions of both of the algae extracts for HSV-1, as evidenced by significant reductions in the yields of viral titers in HeLa cells treated with these preparations. Likewise, the expression of HSV-1 proteins was reduced with the >10 kDa fraction, as shown in Figure 7C. Regarding HSV-2, the *D. antarctica* > 10 kDa fraction displayed a more substantial capacity to inhibit virus yield as compared to the <10 kDa fraction. However, the >10 kDa and <10 kDa fractions of *M. pyrifera* showed modest inhibition of viral replication for this virus and only when combined displayed strong antiviral activity (Figures 7D,E). The results described above were overall paralleled in western blot analyses, with the >10 kDa fractions from both algae generally displaying higher inhibition of viral protein expression. Again, potent inhibition of HSV-2 protein expression was observed when combining the >10 kDa and <10 kDa fractions of the *M. pyrifera* extract (Figure 7F). Importantly, the antiviral effect described above was not due to reduced cell viability (Supplementary Figure 2). Based on these results, we speculated that the antiviral compounds present in the preparations were protein in nature rather than small molecules. To assess this possibility, proteins were precipitated from the extracts using saturated concentrations of sulfate ammonium, which were then removed from the preparation by dialysis. As shown in Figure 8, the precipitated proteins from both algae extracts displayed significant antiviral activity against HSV-1 (Figures 8A,B) and HSV-2 (Figures 8E,F), indicating potent antiviral activity by these biomolecules. Interestingly, the supernatants of the precipitated proteins, which were dialyzed to remove the sulfate ammonium, also displayed significant antiviral activity (Figures 8A,B,E,F), suggesting that there are also active antiviral compounds present in this fraction.

Because the precipitated proteins of the algae extracts overall showed more potent antiviral activity than the whole extracts, higher dilutions of the protein fractions were evaluated against HSV-1 (Figures 8C,D) and HSV-2 (Figures 8G,H). Interestingly, we observed that the antiviral activity of the precipitated proteins extended to a concentration of 0.001 mg/ml for the *D. antarctica* extract, while only 0.1 mg/ml for the *M. pyrifera* extract (Figures 8C,D,G,H).

### Therapeutic Potential of the Algae Extracts Assessed in an HSV-1 Skin Infection Model

**Figure 9:**
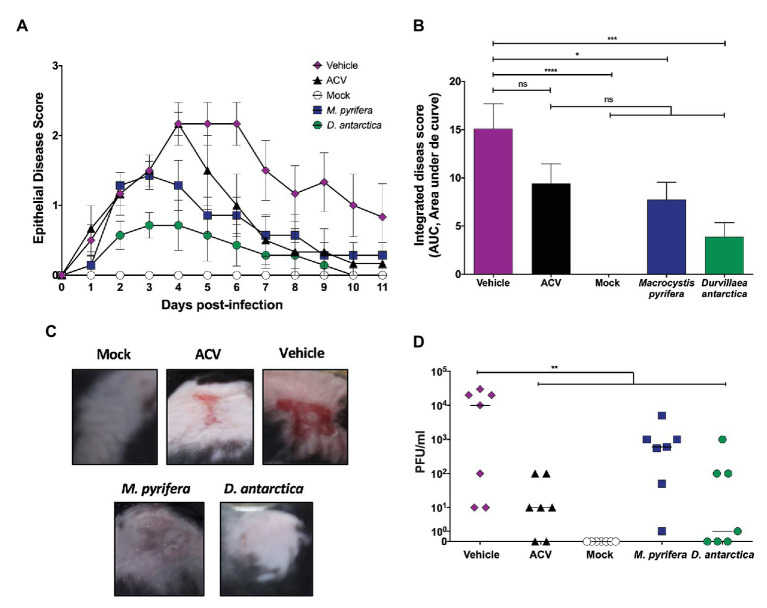
Therapeutic effect of the *M. pyrifera* and *D. antarctica* algae extracts in an HSV-1 skin infection model. Mice were infected with HSV-1 in the flank skin and treated 24 h after infection with the algae extracts formulated in a hydrogel preparation at 5 mg/ml twice a day. The vehicle, commercially available 5% acyclovir cream, and mock-infected animals were included as controls. **(A)** Skin clinical scores were assessed daily for each animal. **(B)** Area under the curve (AUC) values, which integrates the clinical skin scores and their duration between day 0 and day 11 post-HSV-1 infection. **(C)** Representative images of skin lesions at day 5 post-infection with HSV-1. **(D)** Viral titers (PFU/ml) in 200 mg of skin biopsies obtained at day 5 post-infection. Data shown are means ± SEM of two independent experiments (*n* = 7/group). The data were analyzed using one-way ANOVA with Bonferroni’s post-test. ^****^p < 0.0001; ^***^p < 0.001; ^**^p < 0.01; and ^*^p < 0.05.

Given the *in vitro* results reported above, we sought to test the antiviral potential of the extracts in a mouse skin infection model using HSV-1 (Petro et al., 2015). Animals were depilated in the flank, their skin slightly scorched consistently with a nail file, and then infected with HSV-1. Animals were evaluated daily for 10 consecutive days for herpetic cutaneous manifestations. To better mimic the natural course of treatment of cutaneous herpetic lesions in people, the animals were treated for the first time with the algae extracts 24 h after infection. The algae extracts were formulated at 5 mg/ml in the pharmaceutically-based hydrogel vehicle hydroxymethyl cellulose and applied twice a day to the animals, separated by 8 h. Commercially available 5% acyclovir cream was used as a control. The vehicle of the formulations, without the algae extracts, was used as a negative control (vehicle). Mock-infected animals were also included as controls to follow the natural healing process after skin depilation and abrasion. Daily clinical scores detailed in the materials and methods section were plotted for each animal group (Figure 9A), and the area under the curve (AUC), which considers both, the disease score and the healing time, was calculated and plotted separately (Figure 9B). As expected, the animals treated with acyclovir displayed lesser clinical scores than vehicle-treated animals, as well as shorter periods of disease manifestation, which translated into a lower AUC value, as compared to the vehicle group. However, this difference was not statistically significant, which parallels this drug’s effects in some persons (Leflore et al., 2000; Evans et al., 2002). Importantly, animals treated with the *M. pyrifera* extract displayed significantly reduced disease scores and AUC value, as compared to the animals treated with the vehicle (Figures 9A–C). Animals treated with the *D. antarctica* extract also displayed lesser disease scores and AUC value than vehicle-treated animals and acyclovir-treated animals (Figures 9A–C).

Furthermore, the treatments with the algae extracts reduced the viral loads in the infected skin, as shown in Figure 9D. On the other hand, as expected all animals had equivalent amounts of viral DNA in the dorsal root ganglia associated to the site of infection, evidencing that all animals were similarly infected, as the application of the extracts was therapeutic and non-prophylactic and was applied 24 h after infection with HSV-1 (Supplementary Figure 3).

## Discussion

Although numerous antiviral drugs are available for the treatment of HSV infections, such as acyclovir, penciclovir, and famciclovir, which reduce the severity of different forms of HSV-related diseases, such as encephalitis and keratitis, these compounds are somewhat poorly effective at decreasing the cutaneous manifestations produced by these viruses (Leflore et al., 2000; Evans et al., 2002). Indeed, when used topically, these drugs only reduce the duration of the herpetic lesions and associated pain in 1–3 days, when compared to the untreated groups (Emmert, 2000). Importantly, virus isolates that are resistant to these compounds occur, hindering their use (Bacon et al., 2002). Although ganciclovir, cidofovir, and foscarnet can be used against these drug-resistant variants, they can produce numerous adverse effects in the patients (Deray et al., 1989). Thus, better alternatives are needed, and novel options are emerging, such as the combination of acyclovir with hydrocortisone for topical use (i.e., Xerese®, Medivir; Strand et al., 2012), 10% docosanol formulated as a topical cream (i.e., Abreva®, Avanir; Pope et al., 1998), and a zinc oxide-based cream (i.e., Novitra®, Boericke and Tafel; Godfrey et al., 2001). However, their efficacy remains somewhat modest, with healing time improvements for oral HSV lesions within 18 h to 1.5 days in most cases (Godfrey et al., 2001; Sacks et al., 2001; Woo and Challacombe, 2007).

Importantly, several algae extracts display antiviral effects against HSVs, and recent studies have focused on characterizing and purifying the bioactive compounds (Álvarez et al., 2020). Some studies have found that the antiviral molecules are proteins, while other carbohydrates (Bleakley and Hayes, 2017; Lafarga et al., 2020). Interestingly, polysaccharides such as sulfated fucans and sulfated xylogalactofucan from different types of algae have been shown to inhibit HSV-1 or HSV-2 attachment to host cells (Mazumder et al., 2002; Adhikari et al., 2006; Mandal et al., 2008; Saha et al., 2012). On the other hand, lectins and phycobiliproteins derived from algae have also been shown to have antiviral activities against HSVs (Shahidi and Rahman, 2018; Hwang et al., 2020). A well-characterized alga protein with anti-herpetic activity is Griffithsin (GRFT), a lectin isolated from the marine red alga *Griffithsia* sp., which can interact with specific glycan structures present in glycoproteins on the surface of the viral envelope and that interferes with virus binding to the surface of target cells (Mori et al., 2005; Levendosky et al., 2015; Lusvarghi and Bewley, 2016; Derby et al., 2018).

Unlike most algae-derived extracts or algae compounds with antiviral activity mentioned above, which mainly report antiviral activities based on blocking the binding of virus to the cell surface (Levendosky et al., 2015; Lusvarghi and Bewley, 2016; Mahomoodally et al., 2019), the results obtained herein suggest that the *M. pyrifera* and *D. antarctica* extracts mainly block the replication cycle of HSV at steps that are posterior to viral entry, because adding these extracts after infection and removing them before the synthesis of new viral particles was effective at reducing virus yields in the cell cultures. Nevertheless, other antiviral activities are likely also present in the extracts, as the *M. pyrifera* extract was able to reduce the binding of HSV-2 to the cell surface. Furthermore, significant antiviral activities were observed in the different alga extract fractions when separating the compounds by size or when separating the proteins. Interestingly, we observed that a significant portion of the extracts’ antiviral activity was concentrated in the >10 kDa fraction, which suggests that the bioactive compounds are more likely proteins than peptides. This notion is in line with the fact that precipitated proteins displayed significant antiviral activity. Noteworthy, the antiviral activity of the *M. pyrifera* extract against HSV-1 was mostly evident when combining the >10 kDa and <10 kDa fractions, suggesting that the bioactivity of this extract against this particular virus likely relies on more than one component in the extract. Another interesting finding was that the supernatants of the precipitated proteins retained antiviral activity, suggesting again that there may be several antiviral bioactive compounds in the extracts. Nevertheless, it is possible that the protein or proteins with antiviral activity in the extracts did not fully precipitate during the experimental conditions described herein.

It is important to note that the findings described above, together with different capacities of the extracts to interfere with the binding of HSV-2 to the cell surface, suggest distinct mechanisms of action for the extracts studied herein against HSV-1 and HSV-2, altogether evidencing that there are differences between both viruses in terms of their susceptibility to these extracts. Interestingly, there are some previously reported aspects that distinguish these viruses, such as the viral glycoproteins involved in their entry into target cells, with HSV-2 infection occurring independently of glycoprotein C, while this protein is necessary for HSV-1 infection (Herold et al., 1991). On the other hand, while the production of nitric oxide is downregulated in HSV-1-infected cells, it is upregulated after HSV-2 infection, and DNA fragmentation due to apoptosis is blocked by HSV-1 but not by HSV-2 (Jerome et al., 1998; Thakur et al., 2000). Related to disease manifestations, while HSV-1 is frequently associated with primary infections of the lips and eyes and viral reactivations mostly occur from the trigeminal ganglia, HSV-2 is more frequently associated with genital infections and reactivations from the dorsal root ganglia (Whitley and Roizman, 2001; Suazo et al., 2014). Given the findings reported in our study, it will be interesting to identify in future studies the mechanisms of action of these extracts and potentially identify new aspects that differentiate these viruses.

The data reported herein show that the topical application of the algae extracts over a skin area infected with HSV-1 significantly reduced the clinical score associated with the viral infection, as well as the replication of the virus in the skin of the mice. Importantly, we used a treatment scheme that started 24 h after infection to better mimic real-life conditions, with topical antivirals being applied therapeutically, rather than prophylactically. We chose a treatment scheme based on two applications per day, which better mimics the results usually obtained with acyclovir in humans, which is somewhat poorly efficacious (Spruance et al., 1982; Spruance, 1993; Emmert, 2000; Leflore et al., 2000; Bacon et al., 2002; Evans et al., 2002; Moomaw et al., 2003; Woo and Challacombe, 2007). Notably, under these conditions, we observed that the *M. pyrifera* and *D. antarctica* extracts significantly reduced the clinical scores of herpetic skin lesions as compared to vehicle-treated mice and that these scores were lesser than those obtained with acyclovir. These results suggest that the algae extracts perform better than acyclovir against HSV-1 skin lesions, or eventually similarly with less frequent applications than acyclovir. Noteworthy, the *D. antarctica* extract showed slightly better efficacy than the *M. pyrifera* extract, both in terms of lower AUC values for the clinical scores and significantly lesser viral loads in the skin. The reduced amount of virus detected in the skin of the animals treated with *D. antarctica* extract was not due to reduced infection, as HSV-1 loads were equivalent in the dorsal root ganglia of all the groups. An interesting finding was that although the *M. pyrifera* extract significantly reduced the clinical score AUC values unlike acyclovir, it did not significantly reduce the viral loads in the skin, contrary to acyclovir. This finding suggests that the clinical scores associated with skin lesions seems not necessarily directly linked to the viral loads in this tissue.

Taken together, the findings reported in this study indicate that the aqueous extracts from *M. pyrifera* and *D. antarctica* exhibit significant antiviral activity against acyclovir-sensitive and acyclovir-resistant HSV-1 and HSV-2 and that either extract formulated topically can reduce the severity of skin lesions produced by HSV-1 to a better extent than acyclovir. Importantly, both of these algae are regularly consumed as food or food supplements in some countries, suggesting that they are safe to use. Given the favorable results reported herein, it will be interesting to assess the therapeutic potential of these formulations in clinical settings in individuals with recurrent HSV lesions produced by either acyclovir-sensitive or acyclovir-resistant infections.

## Data Availability Statement

All data supporting the conclusions in the manuscript are included in the article/Supplementary Material. Inquiries can be directed to the corresponding author.

## Ethics Statement

The protocol for primary cultures of human gingival fibroblasts derived from the tissue of the masticatory mucosa in the retromolar area of healthy adults was reviewed and approved by the Institutional Ethics Committee protocol #60823025. The animal study was reviewed and approved by the Institutional Ethics Committee for Animal and Enviromental Care protocol #170416002.

## Author Contributions

All authors contributed to the writing and editing of the manuscript. EC, LD, NC, DA, MF, and AH contributed with experiments. CA-M and PG contributed to the design of the study. All authors contributed to the article and approved the submitted version.

### Conflict of Interest

Patents related to the results described in this study have been submitted to PCT.
