# Supplementary material for: Anti-herpetic Activity of Macrocystis pyrifera and Durvillaea antarctica Algae Extracts Against HSV-1 and HSV-2
Source: Front Microbiol. 2020 Sep 11;11:2006. doi: 10.3389/fmicb.2020.02006 (PMC7516053; doi:10.3389/fmicb.2020.02006)
Supplement: Supplementary file 1 [file Data_Sheet_1.docx]

**SUPPLEMENTARY FIGURE 1**

**Supplementary Figure 1. Determination of the 50% cytotoxic concentration (CC_50_) of the *Macrocystis pyrifera* and *Durvillaea antarctica* algae extracts.** Cells were incubated with serially-diluted algae aqueous extracts starting at 125 mg/mL and the cell viability was evaluated at 24 h post-treatment using a resazurin-based assay (alamarBlue®). **(A)** Viability curve showing the CC_50_ of the *Macrocystis pyrifera* extract in HeLa cells. **(B)** Viability curve showing the CC_50_ of the *Durvillaea antartica* extract in HeLa cells. **(C)** Viability curve showing the CC_50_ of the *Macrocystis pyrifera* extract in human gingival fibroblasts. **(D)** Viability curve showing the CC_50_ of the *Durvillaea antartica* extract in human gingival fibroblasts. Data shown are means ± SEM of three independent experiments.

**SUPPLEMENTARY FIGURE 2**

**Supplementary Figure 2. Maximum non-toxic dose (MNTD) of the *Macrocystis pyrifera* and *Durvillaea antarctica* size-fractionated extracts*.*** (**A**) MNTD in HeLa cells treated with the *Macrocystis pyrifera* size-fractionated extract. (**B**) MNTD in HeLa cells treated with *Durvillaea antarctica* size-fractionated extract. UT: untreated cells; EtOH: cells treated with 70% ethanol. Data shown are means ± SEM of three independent experiments. The data were analyzed using one-way ANOVA and Dunnett’s multiple comparisons test; **** p<0.0001, * p<0.05*.*

**SUPPLEMENTARY FIGURE 3**

**Supplementary Figure 3. Viral loads (qPCR) in the dorsal root ganglia of HSV-1-infected and *Macrocystis pyrifera* or *Durvillaea antarctica* algae extract-treated animals.** Viral genome copies (DNA, *UL30* gene) were determined by qPCR in the dorsal root ganglia of HSV-1-infected animals at 11 days post-infection. Data shown are means ± SEM (n=3-4/group). The data were analyzed using one-way ANOVA with Bonferroni’s post-test; No significant differences were observed between groups*.*
